# Vascular inflammation in aging

**DOI:** 10.18632/aging.101653

**Published:** 2018-11-15

**Authors:** Indranil Biwas, Alireza R. Rezaie

**Affiliations:** 1 Cardiovascular Biology Research Program, Oklahoma Medical Research Foundation, Oklahoma City, OK 73104, USA

**Keywords:** cardiovascular disease, inflammation, aging, damage associated molecular patters, polyphosphate

Cardiovascular disease (CVD) is the leading cause of morbidity and mortality worldwide. Vascular inflammation is a crucial factor in the development of age-related CVD including atherosclerosis, thrombosis and heart failure. Accumulating evidence suggests that aging is associated with chronic low-grade inflammation, termed sterile-inflammation, which can contribute to sustained decline in cardiovascular function [1]. The origin of sterile inflammation is not well understood. Recent results have indicated that ischemia, injury, hormonal imbalance or cellular stress may lead to sterile inflammation through release of endogenous damage associated molecular patterns (DAMPs) such as DNA, RNA, histones, and high mobility group box 1 (HMGB1). These biomolecules can initiate and propagate noninfectious sterile inflammatory responses by binding and signaling through pattern recognition receptors [i.e., receptor for advanced glycation end products (RAGE) and toll-like receptors (TLRs)] which are expressed on the surface of cells of the innate immune and vascular systems [2]. DAMP-mediated sterile inflammation can induce endothelial cell activation and endothelial-leukocyte interaction thus culminating in endothelial cell injury, disruption of endothelial barrier function and transmigration of leukocyte into the subendothelial tissues. The activation of endothelial cells by DAMPs also endows a procoagulant phenotype for the vasculature thus promoting thrombin generation, which in turn can augment inflammation [3]. This amplification loop mediated by inflammation and coagulation must be tightly regulated and a defect in the molecular players of either anti-inflammatory or anti-coagulant regulatory pathways can cause vascular dysfunction, accelerating CVD progression. There is increasing evidence, which suggests that these regulatory pathways may be downregulated by aging, thereby contributing to the development of age-related CVD [4]. DAMP-mediated activation of inflammation and coagulation also have an impact on platelet reactivity and function. A plethora of research on myocardial dysfunction and atherosclerosis has focused on the role of platelets on the age-related CVD. Platelets play critical roles in the development of arterial thrombus formation and existing antiplatelet therapy (i.e., aspirin and clopidogrel) has been shown to be effective in treating cardio-arterial diseases. Previous studies have demonstrated that platelets also express pattern recognition receptors and are capable of exerting innate immune responses. The role of sterile inflammation on platelet activation is not extensively studied. It is of interest to note that activated platelets also release HMGB1, which can activate coagulation and inflammation. Moreover, recent results have indicated that platelets are a rich source of polyphosphate which is known to augment coagulation and inflammatory pathways via interaction with several coagulation factors and cell surface receptors [5,6]. We recently demonstrated that platelet-derived polyphosphates also activate endothelial cells through simultaneous interaction with both RAGE and P2Y1 receptors, thereby inducing potent inflammatory responses in in vitro and in vivo models [7]. These studies showed that polyphosphates can bind HMGB1 (also histones) to dramatically amplify inflammatory signaling responses in endothelial cells through inducing the activation of NF-kB, the expression of cell adhesion molecules and facilitating activated leukocytes-endothelial cell interactions. Recent research from our lab also demonstrated that platelet-derived polyphosphate by itself and in complex with HMGB1 mediates von Willebrand Factor (VWF) release from endothelial cells, possibly contributing to regulation of coagulation and inflammation [8]. In support of this, we observed that perfused platelets could interact with polyphosphate-HMGB1-treated endothelial cells resulting in platelet-string formation. This interaction was mediated through polyphosphate-HMGB1-mediated VWF release since it was inhibited by an anti-GPIbα antibody [8]. Thus, polyphosphate plays a key role in activation of both coagulation and inflammation. However, the role of polyphosphate on the development of CVD has not been studied. In light of a key role for DAMPs in inducing sterile inflammation and age-related enhanced hypercoagulability that is associated with increased platelet activation, there is a possibility that an age-related increase in the basal plasma levels of polyphosphate could contribute to chronic inflammation and increased risk of CVD. Thus, we believe that it will be worth studying the involvement of polyphosphate together with HMGB1 (or histones) in age associated CVD.
